# High sensitivity but low specificity of the risk factors and symptoms questionnaire in diagnosing female genital schistosomiasis among sexually active women with genital lesions in selected villages of Maswa District, North-Western Tanzania

**DOI:** 10.1371/journal.pntd.0012336

**Published:** 2024-08-12

**Authors:** Gladys Mbwanji, Edgar Ndaboine, Amina Jumanne Yusuf, George Kabona, Boniface Marwa, Humphrey D. Mazigo

**Affiliations:** 1 Department of Parasitology, School of Medicine, Catholic University of Health and Allied Sciences, Mwanza, Tanzania; 2 Bugando Medical Centre, Mwanza, Tanzania; 3 National Neglected Tropical Disease Programme, Ministry of Health, Dodoma, Tanzania; 4 Simiyu Regional Hospital, Ministry of Health, Simiyu, Tanzania; 5 School of Public Health, Dean’s Office, Catholic University of Health and Allied Sciences, Mwanza, Tanzania; George Washington University, UNITED STATES OF AMERICA

## Abstract

**Background:**

The diagnosis of Female Genital Schistosomiasis (FGS) which is a clinical feature of urogenital schistosomiasis caused by *Schistosoma haematobium* is challenging, especially in primary healthcare facilities characterized by low resources which are dependent by the majority of the FGS endemic communities. To facilitate and improve diagnosis in these settings, a simple risk factors and symptoms tool has been developed to help healthcare workers at primary healthcare facilities identify and manage FGS cases. However, the sensitivity and specificity of the tool are not known. Therefore, the objective of this study was to assess the performance of risk factors and symptoms tools in diagnosing FGS in adolescent girls and women of reproductive age in selected villages of north-western Tanzania.

**Methods:**

A community-based analytical cross-sectional study was conducted among 347 women aged 18–49 years in Maswa District, north-western Tanzania. A single urine sample was collected from each participant and screened for *S*. *haematobium* eggs using a urine filtration technique. Consenting participants (n = 177), underwent thorough speculum examination by trained gynaecologists using a digital portable colposcopy to capture images of the cervix and vagina. All the captured pictures were examined independently by two pairs (2 gynaecologists in each pair) of qualified obstetricians and gynaecologists. A descriptive analysis and logistic regression were used to demonstrate the prevalence, symptoms, and risk factors of FGS.

**Results:**

The mean age of 347 women enrolled in the study was 30 years (Standard Deviation (SD) ±7.7) and the prevalence of women with symptoms suggestive of FGS was 15.8% (95% CI; 10.8%- 22.0) by colposcope and 87% (95% CI; 83.0%-90.4%) using the risk factor and symptom checklist. The overall sensitivity, specificity, positive and negative predictive value of symptoms and risk factors checklist tool for diagnosing FGS schistosomiasis (≥7 score points) using colposcope as a reference test were 85.7% (95%CI; 80.6%- 90.9%), 8.7% (95%CI; 4.6%-12.9%), 15.0% (95%CI; 9.7%-20.3%) and 76.5% (95%CI; 70.2%-82.7%). Multivariate analysis showed that female genital schistosomiasis using a risk factor and symptom checklist was associated with fetching water in contaminated fresh water (aOR:21.8, 95%CI;2.8–171.2, P <0.003), self-reported pelvic pain (aOR:5.3, 95%CI; 1.1–25.9, P< 0.04) and having any urinary symptoms (aOR:12.2, 95%CI; 1.5–96.3, P<0.018). Urine microscopy results were available for 345 participants, of these, 3.5% (12/345) (95% CI; 1.8%-6.0%) were positive for *S*. *haematobium* infection.

**Conclusion:**

Female genital schistosomiasis and urinary-related symptoms are common in the current study population. The risk factor and symptoms checklist for diagnosis of FGS achieved high sensitivity but low specificity for women who scored ≥7 points using colposcope as a reference diagnostic test. At present, the call to integrate FGS into the reproductive health services for women has received much attention, however, the diagnostic part of FGS remains a challenge, thus there is a need to continue evaluating this tool in different population and age structures in endemic areas.

## Author summary

Female genital schistosomiasis (FGS) is a genital manifestation of a waterborne parasitic disease caused by *Schistosoma haematobium*, however, it is mostly neglected. FGS diagnosis is still a challenge due to the lack of reference standards for its diagnosis. Colposcope has been used as a standard for diagnosing FGS, it shows the FGS clinical findings on the cervix and vagina. However, FGS diagnosis by colposcope has not been feasible in most rural endemic settings due to its unavailability, low level of knowledge of its use for diagnosing FGS, and low FGS knowledge among healthcare workers. Thus, there is a need to develop an alternative cheap diagnostic tool to be used in endemic areas for identifying women and adolescent girls at high risk of having FGS lesions and referring them for colposcopy examination. This call has been given more weight by the WHO in its roadmap plan for ending Neglected Tropical Diseases (NTD) by 2030. In response to this call, this study determined the sensitivity and specificity of the risk factors and symptoms checklist (variables), and the prevalence of FGS by both colposcope and risk factors and symptoms checklist using a score point of 7 as a cut-off point to suspect FGS. Furthermore, we also determined the prevalence of *S*. *haematobium* infection using a urine filtration technique and determined the symptoms and risk factors of urogenital schistosomiasis among women in selected villages of Maswa district, north-western Tanzania. The findings of this study demonstrated a high prevalence of FGS in the current study population using both methods/diagnoses. There is a need for further evaluation of the performance of the risk and symptoms questionnaire to improve its performance. Furthermore, there is a need to integrate the FGS services into a wide spectrum of reproductive health services, to allow women with urogenital symptoms to receive treatment.

## Introduction

Female genital schistosomiasis (FGS) is a chronic gynecological disease of girls and women of reproductive age caused by *Schistosoma haematobium* [1,2]. There is a significant gap in accurate epidemiological data on the burden of FGS in endemic areas. The available estimates indicate that two-thirds of women infected with *S*. *haematobium* can lead to FGS in 33–75% of these women [3] and another estimate indicates that FGS affects at least 16 million women [2]. Even in the absence of ova excretion in women, 23–41% of them are found to have genital schistosomiasis [4,5]. FGS has been implicated as a major cofactor in poor women’s reproductive health. FGS can cause infertility, menstrual and pregnancy complications, genital lesions, pain and bleeding from intercourse, anemia, genital itching, and pelvic pain [6]. The typical biological changes, such as sandy patches, chronic inflammation, vascularization, and pathological blood vessels, can make women more susceptible to infections such as HIV, decreased fertility, and increased risk of miscarriage [1,6,7]. The genital-related symptoms tend to mimic Sexually Transmitted Infections (STIs), commonly resulting in misdiagnosis. Despite causing significant suffering to millions of women and girls, FGS has been neglected [1]. For instance, even though, Tanzania is second in sub-Saharan Africa country for having the highest prevalence of schistosomiasis, (with two-thirds of the cases estimated to be caused by *S*. *haematobium* [8], FGS is mostly neglected. Studies on FGS are very limited and the limited evidence indicates that, in north-western Tanzania, the prevalence ranges from 0–12% [9]. In Tanzania, FGS is not considered a priority health problem in both national and local policies and programs [10].

Female genital schistosomiasis diagnosis includes urine microscopy, gynecological examination by speculum/colposcope, biopsy of genital tissue, and molecular diagnosis of genital samples collected at home or clinic [7,11–13], all of which have challenges. In addition, FGS diagnosis is challenged by the need for gynecological examination (ideally with specialized equipment) and a high level of specialized training among health workers [14]. All of these are not available in most of the health facilities in rural endemic areas. While it is acknowledged that the use of highly specialized equipment may not be accessible for FGS diagnosis in primary healthcare facilities in endemic areas [15], developing a simple diagnostic algorithm [16] with relevant questions to be used by healthcare workers could help in identification of at-risk adolescent girls and women, who could then be referred to higher-level facilities for further assessment and diagnosis. However, for healthcare workers to adapt to this, there is a need to be trained on how to ask comprehensible questions about FGS symptoms (history taking about involvement in risk environment, vaginal discharges, urinary incontinence, irregular vaginal bleeding, postcoital bleeding, lower abdominal pain, infertility and previous treatment history and outcomes in routine clinical practice) when women and adolescent girls seek for FGS diagnosis in healthcare [14].

The WHO has called for the development of easy-to-use diagnostic tests for the diagnosis of FGS as a priority in its 2021–2030 roadmap for Neglected Tropical Diseases (NTDs) elimination to attain Sustainable Development Goal (SDG) number three- health for all, no one is left behind [17]. The countdown project in Nigeria and Liberia developed a manual that aims to assist healthcare workers in diagnosing and treating women with FGS by using the FGS symptoms and risk factors checklist (S1 Information) [16,18]. However, there is limited evidence on the performance (sensitivity and specificity) of this tool in diagnosing or identifying women suspected to have FGS-related symptoms and referred for gynecological examination. This calls for the need to further evaluate the performance of this tool in different populations in schistosomiasis endemic areas. Therefore, the overall aims of the current study were to assess (i) the specificity and sensitivity of the FGS risk factors and symptoms checklist in identifying women with suggestive symptoms of FGS compared to colposcope and (ii) to determine the prevalence and risk factors of urogenital/urinary schistosomiasis among women and adolescent girls and (iii) to assess the prevalence of FGS among sexually active women using the digital colposcope. We hypothesized that the FGS symptoms and risk factors checklist had higher specificity and sensitivity in diagnosing FGS among sexually active women using the digital colposcope as a reference diagnostic test.

## Methodology

### Ethics statement

The ethical approval was obtained from the National Ethical Review Committee, under the National Institute for Medical Research (NIMR) (Certificate number NIMR/HQ/R.8a/Vol.1X/4072). Further permissions to conduct the study were obtained from the local authorities (regional, district, ward, and village leaders) before the study was implemented. Information about the study was provided to potential participants before enrolling in the study, and the importance of volunteering in the study and signing informed consent forms was emphasized. All participants signed, written informed consent (or fingerprint for women who could not read and write) before the investigation was done.

The study was implemented according to the principle stated in the Declaration of Helsinki and the principle of the International Conference on Harmonization Guidelines for Good Clinical Practice (GCP). Participants’ details were kept confidential and each participant was allocated a unique number. Participants were informed about their right to withdraw from the study at any time if they wished to do so. Women were also asked to consent to the colposcope examination. All Colposcopy images obtained were de-identified: Study participants diagnosed with other diseases such as STIs were treated on-site and those with other diseases which could not be treated at the data collection sites were counseled and referred to a nearby health facility for further evaluation and treatment.

### Study population and area

Maswa district is one of the six district councils of the Simiyu region. The district has 36 wards and 120 registered villages. The district is known to be endemic for urogenital schistosomiasis [19]. According to the 2022 National Population and Housing Census, Maswa district has a population of 427,864 people, 208,255 males and 219,609 women [20]. Overall, 80% of the population is involved in agriculture and livestock keeping [8]. The main sources of water are irrigation schemes, man-made dams, springs, small rivers, and lakes.

The district has 48 health facilities that conduct preventive and curative services. One of the common diseases treated at the district health facilities is schistosomiasis [19]. Maswa district has seasonal rivers and swamps used for paddy farming which provide conducive environments for *S*. *haematobium* transmission [19]. Most of the north-western settings are traditionally endemic to *S*. *haematobium* [8].

### Study design, inclusion, and exclusion criteria

An analytical cross-sectional survey was conducted among 347 adolescent girls and women of reproductive age in selected villages of Maswa district. The selection of the villages was based on the prevalence of urogenital schistosomiasis (based on microscopic examination of urine samples) among school-aged children [20].

The study recruited and enrolled sexually active adolescent girls and women aged 18–49 years with written/witnessed informed consent, able to produce urine for urine filtration test, willing to undergo comprehensive speculum /colposcopy examination, permanent residence of the selected communities/villages and living/working close to freshwater sources for the past two or more years were recruited into the study. Pregnant women and adolescent girls (<18 years) were excluded from this study.

### Sample size and calculation

Using a single proportion sample size formula at a 95% confidence interval and tolerable error of 0.03, the total sample size calculated was 386. Considering the refusal to participate and loss to follow-up, 5% of the sample was added. The sample size was calculated based on the mean prevalence of FGS reported by a recent cross-sectional study conducted in north-western Tanzania, a prevalence of 5% among women aged 18–50 years [9].

### Recruitment procedure

In each village, the community engagement team together with a community leader invited village/community members to participate in the study by either blowing a whistle to catch people’s attention or by inviting women individually using community healthcare workers. This was followed by playing local Kiswahili songs to sensitize the communities. Girls and women who responded to the invitation and met the inclusion criteria were invited to participate in the study.

Women with informed consent, who produced 10mls of urine were recruited and underwent thorough speculum examination by a trained gynaecologist using a portable colposcopy (EVA MobileODT, Israel) to capture images of the cervix and vagina [21]. Urine samples were collected between 10 am to 2 pm due to the circadian pattern of egg excretion [22] and processed using urine filtration technique on the same day as the colposcopy examination.

### Data collection

A data collection tool (pre-tested questionnaire) was developed to collect all information needed to answer the study questions. Trained research assistants conducted a face-to-face structured interview using pretested questionnaires to collect data and fill the questionnaire.

The questionnaire was used to collect social-demographic characteristics (age, education level, occupation), reported history of praziquantel use, the FGS related symptoms (genital bleeding, pelvic pain, genital ulcerations, genital discharge, irregular menses, and pains during urination), and FGS-related risk factors (exposure activities to freshwater, distance from freshwater, other sources of water for domestic activities). To ensure quality, collected data were checked daily and for any missing data, participants were re-contacted to provide the missing information. For variables that information was not obtained the variables with missing information were dropped during the analysis. Otherwise, analysis would have been done to examine whether participants with missing data appeared to be systematically different from other participants, to assess the risk of bias in the results. A complete records analysis approach would be used to deal with missing data.

### Parasitological examination of urine samples for *S*.*haematobium* eggs

From each study participant, a single urine sample was collected between 10 am to 2 pm and visually examined for the presence of macro-haematuria using a color chart, for microhaematuria and the presence of *S*. *haematobium* eggs a urine reagent strips (Hemastic, Siemens Healthcare Diagnostics GmbH, Germany) and urine filtration technique were used [23]. For quality control, 10% of positive slides and negative slides were re-examined for the presence of *S*. *haematobium* eggs by an expert microscopist. The results of micro-haematuria were recorded as positive and negative.

Timeline of different activities in this study was done on the same day starting with screening women for eligibility criteria, participants who were eligible and consented were recruited in the study. A questionnaire that contained a risk factors and symptoms checklist with score points to determine the presence of FGS by checklist was conducted by trained research assistants. These activities were proceeded by urine sample collection, speculum, and colposcopy examination. The urine filtration method was done by a laboratory technician in the designated laboratory examination room. FGS screening and diagnosis by colposcope and by checklist are explained below.

### FGS suspects by using a checklist

A checklist was used to screen for and diagnose FGS. It consisted of three risk factors and seven symptoms related to FGS (S1 Information). Each risk factor or symptom had a score point depending on the risk of exposure to FGS. A minimum score point of ≥ seven [7] points on the checklist, was used to indicate that a participant had FGS or was suspected to have FGS. The rationale for choosing 7 as a minimum score point was based on increased exposure to selected exposure or history of exposure to multiple risk factors/symptoms that increased the risk of having FGS lesions/symptoms (the cut-off point was considered as a mean cut-off point of multiple exposures.

### FGS diagnosis by visual inspection

The gynecological examination was conducted as described elsewhere [13]. Briefly, all women who consented to speculum examination were sent for speculum and colposcopy examination. Two trained gynaecologists performed the speculum examination, collected swab(s) collection for molecular diagnostics, and colposcopy following the written standard operating procedures (SOPs) for gynecological examination [13]. Examination procedures were explained to participants before taking part in the study. Participants were made comfortable on the examination table and ensured privacy by using a curtain stand or separate room for examination. Participants were positioned in a lithotomy position for a gynecological examination and the gynaecologists performed a speculum examination. The gynaecologists inspected the cervix at low-power magnification (5x to 10x), looking for any obvious areas of abnormality (e.g. sandy patches, rubbery papules). They identified the transformation zone and the original and new squamocolumnar junctions (SCJ). The gynaecologist applied acetic acid to screen for cervical cancer and waited two minutes to allow color changes to develop. The normal squamous epithelium of the cervix is pink. On application of acetic acid, cervical intraepithelial neoplasia (CIN) lesions take on a white colour; due to the increased nuclear proteins and cytokeratins in the cervical epithelium [24]. They observed any changes in the appearance of the cervix, giving special attention to abnormalities close to the SCJ. The trained personnel integrated the findings to make a colposcopy assessment and take a picture for further assessment. They withdrew the colposcope and gently removed the speculum.

Colposcope images were anonymous to ensure privacy and confidentiality. After examination, the gynaecologist explained to the client what they saw during the procedure and, interpreted the findings using a language the participant could understand. For this study, all women who consented to gynaecology examination underwent speculum examination to compare results from the checklist and standard speculum/colposcopy examination for the diagnosis of FGS. All women suspected of any other gynecological condition/diseases were treated at the site and for the disease/condition that needed specialized assessment, participants were referred to a referral or designated health facility for further investigations and management. A colposcopy examination was performed on the same day as the urinary sample collection. All the captured pictures were examined independently by two pairs (2 gynaecologists in each pair) of qualified obstetricians and gynaecologists for diagnosis of FGS.

### Data analysis

Data were double-entered by two trained data entry clerks and cross-checked. A statistician checked for any discrepancies and corrected all errors by referring to the original questionnaires. Only participants with complete data were included in the analysis.

The analysis was performed using Stata version 15 (StataCorp, 2017, Stata statistical software, College Station, TX: StataCorp). Descriptive analysis was used to explore, describe, and summarize the baseline demographic characteristics of study participants. Simple tabulations and cross-tabulation were used to show the prevalence, frequency, and distribution of social-demographic characteristics, symptoms, and risk factors of adolescent girls and women. Regrouping and recoding were done for key independent variables. Categorical variables with data sparsity within groups were regrouped into larger groups to improve statistical stability, whilst being careful to ensure the groupings were logical to minimize loss of detail. Histograms were used to show the distribution of continuous variables (for example age) and assess any data skewness. Continuous variables were presented with mean and Standard Deviation (SD) as appropriate. The chi-square test was used to analyse categorical data.

The risk factors and symptoms of FGS using the score point of ≥7 as the cutoff point were analyzed using the logistic regression. Following univariate analysis, a multivariate logistic model was then fitted using the forward approach, using key parameters that did not exceed 10% of the events. The relationship between binary FGS symptom and risk factors checklist results and risk factors were assessed by using univariate logistic regression and variables with *P*-value <0.05 were considered for multivariate logistic regression analysis. We controlled for confounders by applying multivariate logistic regression as desirable.

Age was used as a priori confounder. Potential confounders were adjusted for and checked for a change in risk ratios from crude risk ratios after adding one confounder at a time. Likelihood Ratio Test (LRT) was used to test for general association and hypothesis testing. All information related to identified confounding factors such as FGS-related symptoms and signs, cervical cancer, urinary tract infections, water contact behaviour, etc, were collected during data collection.

Women who underwent colposcopy examination were considered positive for FGS if they had one of the four genital manifestations described in the WHO FGS atlas [12] namely grainy sandy patches, homogenous yellow sandy patches, rubbery papules and/or abnormal blood vessels [12]. The final diagnosis was done after evaluation from two FGS expert gynaecologists’ pairs through blind assessment. Three categories were used to define FGS from colposcopy images by the gynaecologists (1) the presence of one or more signs of FGS was categorized as positive, (2) the absence of FGS signs as FGS negative, and (3) discordant FGS results are indeterminate between the gynaecologist.

The sensitivity of the diagnostic tool was calculated as the number of true positives (TP) divided by the total number of true positives (TP) and false-negative (FN), and Specificity was calculated as true-negative (TN) divided by the total number of true negative (TN) and false-positives (FP). In the analysis, the positive predictive value as TP/(TP+FP), and negative predictive value as TN/(TN+FN) [25] were also calculated.

## Results

**Fig 1:**
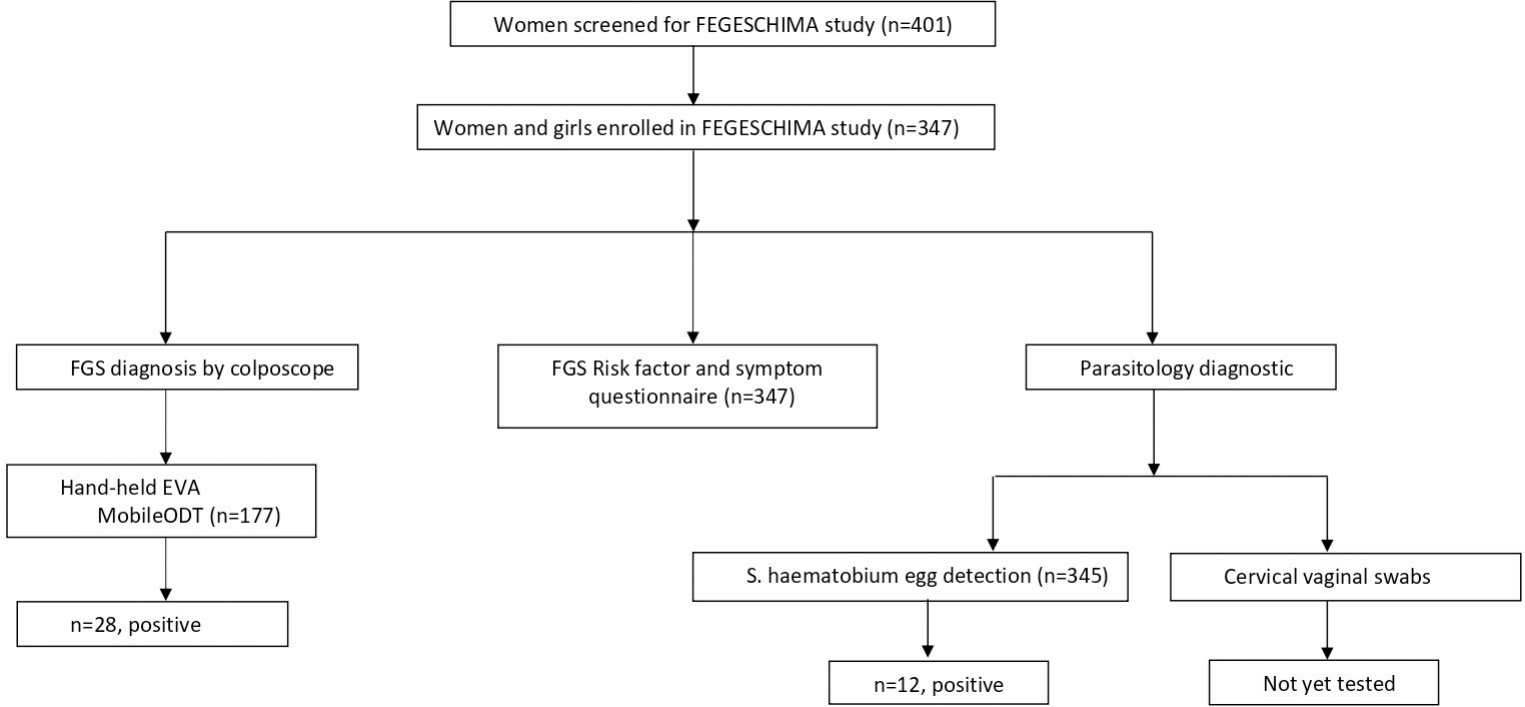
Study flow chart shows the study flow diagram.

Overall, 401 women were screened for eligibility criteria and 347/401(86.5%) women met the inclusion criteria and were enrolled in the study. All 347 enrolled women completed the questionnaire. Hand-held colposcopy was performed on 177/347 (51%) women using handheld EVA MobileODT colposcope. Cervicovaginal swabs were collected from 328/347 (94.5%) women by gynaecologist. A total of 345/347 (99.4%) participants recruited in the study underwent urine microscopy (Fig 1).

### Demographic characteristics of the study participants

A total of 347 sexually active adolescent girls and women, with a mean age of 30 (standard deviation (SD)±7.7) years were recruited into the study. Overall, (90.2%) of participants had no history of using praziquantel drugs. Table 1 summarizes the characteristics of the study participants.

**Table 1:** Baseline characteristic of study participants (N = 347).

Characteristics	Category	Women (n)	Percentage (%)
**Age (SD) mv = 2**	30.8 (±7.7)		
**Age group (years)**	≤ 18	10	2.9
	19–24	73	21.16
	25–29	79	22.9
	30–34	72	20.87
	35–39	53	15.36
	40–44	39	11.30
	45–49	19	5.51
**Religion (mv = 7)**	None	55	16.18
	Muslim	9	81.18
	Christian	276	81.18
**Level of education**	No formal education	83	23.92
	Primary school	213	61.38
	Secondary education	43	12.39
	University/College	8	2.31
**Marital status (mv = 1)**	Married/Cohabiting	268	77.46
	Widow	13	3.76
	Separated/Divorced	22	6.36
	Single	43	12.43
**Employment**	Salary employed	11	3.17
	Self-employed	251	72.33
	Housewife	50	14.41
	Unemployed	27	7.78
	Student	8	2.31
**Contact of water bodies (mv = 3)**	Yes	186	53.60
	No	158	45.53
**Reported use of praziquantel (mv = 3)**	Yes	31	8.93
	No	313	90.20
**Village**	Isanga	127	36.60
	Jija	137	39.48
	Malampaka	83	23.92

Key: mv-missing variables

### Prevalence of urogenital schistosomiasis

Urine microscopy results were available for 345/347(99.4%) participants, the overall prevalence of *S*. *haematobium* infection was 3.48% (95% CI; 1.8%-6%).

### Prevalence of suspected female genital schistosomiasis by the risk factor and FGS symptom checklist

Based on the risk and symptoms questionnaire, 87% were categorized as having FGS. Isanga and Jija villages had the highest prevalence of participants categorized as having FGS. The rationale for choosing seven (7) as a minimum score point was that there was an increased exposure to selected exposure or history of exposure to multiple risk factors that increased the risk of having FGS lesions/symptoms (the cut-off point was considered as a mean cut-off point of multiple exposures).

For the participants who had positive *S*. *haematobium* egg slides, 91.6% (11/12) scored ≥ seven (7) points on the risk factors and symptoms checklist, indicated that they had FGS or were suspected to have FGS and needed further follow-up on examination. Out of 28 participants, 24 (85.7%) of them were diagnosed with FGS by colposcope and had ≥ seven (7) points scored on the risk factors and symptoms checklist. None of the study women was positive for both urogenital schistosomiasis and FGS using the hand-held colposcope.

### Prevalence of female genital schistosomiasis based on digital colposcope

**Fig 2:**
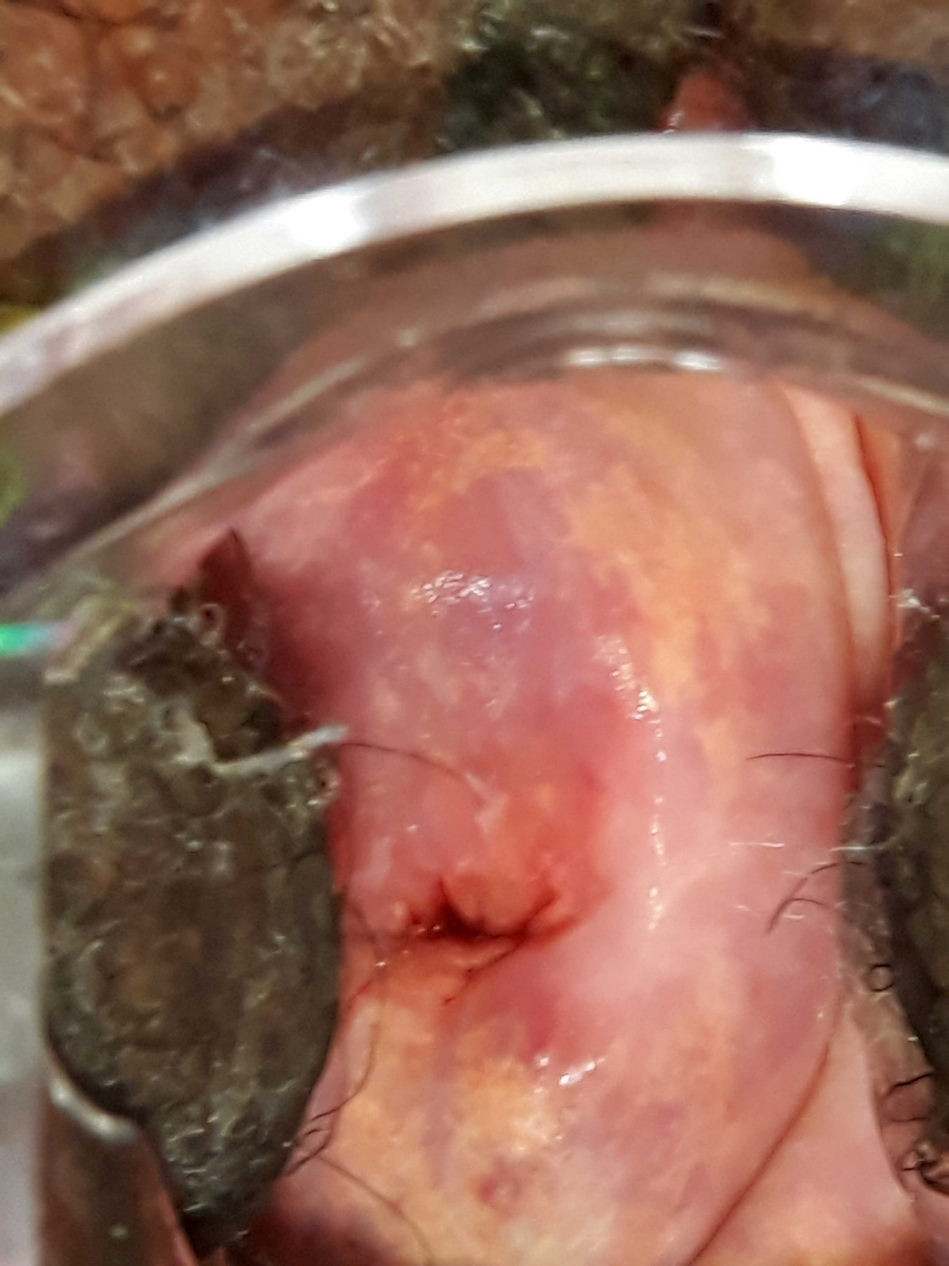
Homogenous sandy patches suggestive of FGS.

**Fig 3:**
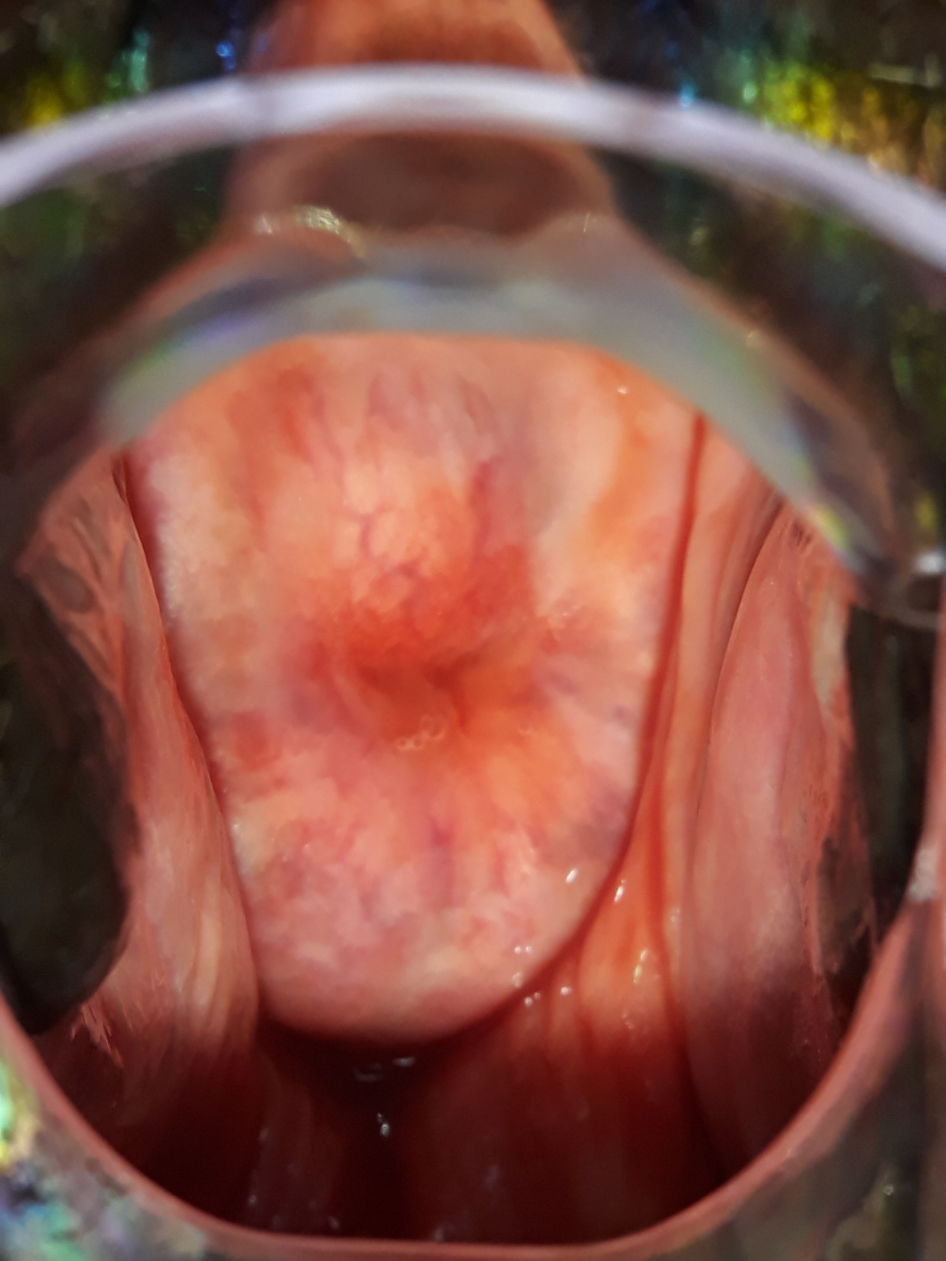
Abnormal blood vessels suggestive of FGS.

**Fig 4:**
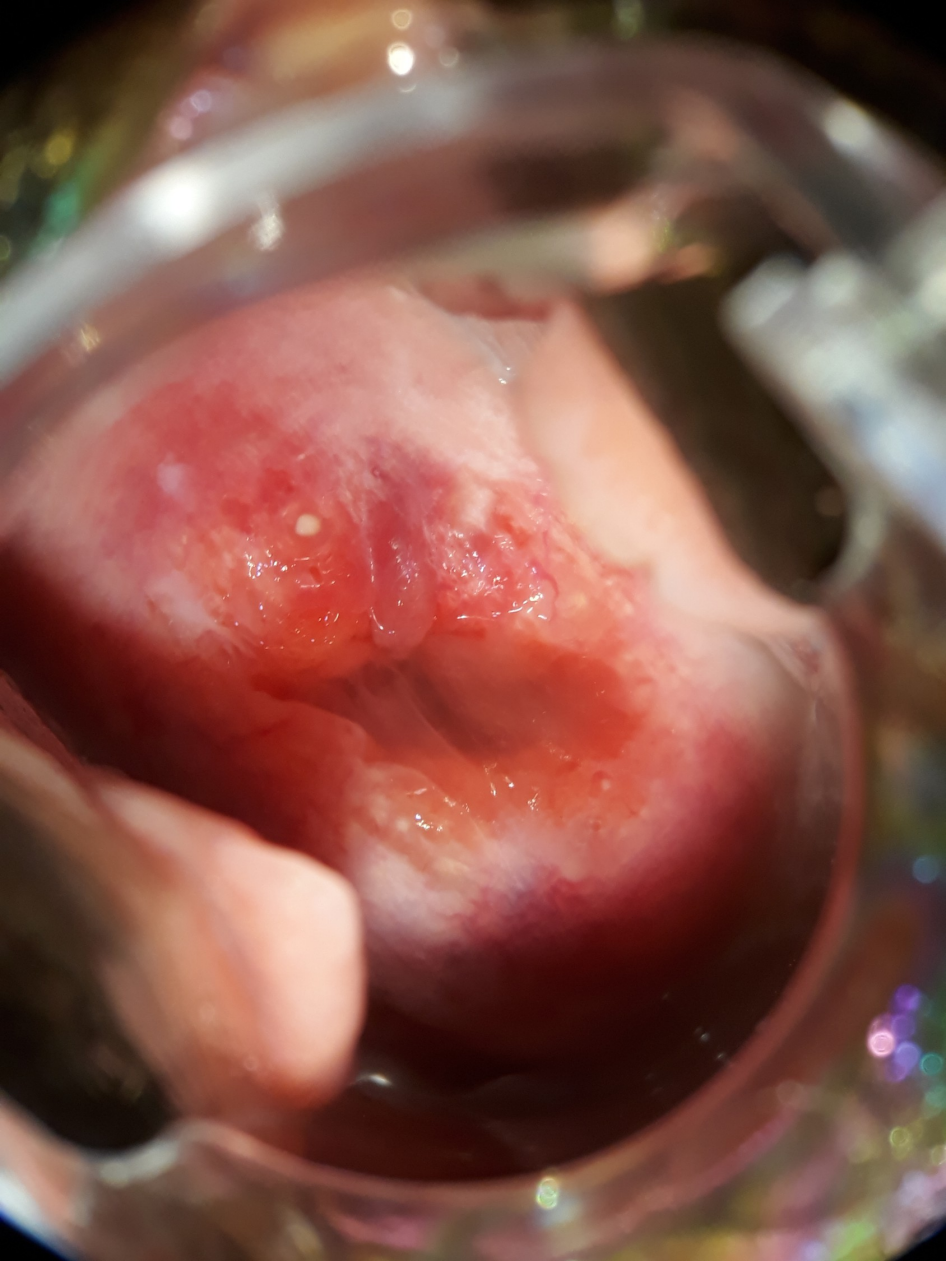
Grainy sandy patches and Rubbery papules.

A total of 177 study participants underwent colposcope examination using a portable digital colposcope (EVA MobileODT). The prevalence of FGS using digital colposcope was 15.8% (28/177; 95% CI: 10.8%-22%). Homogenous yellow sandy patches were observed in 14 (50%) of study participants (Fig 2) and abnormal blood vessels were observed in 7(25%) of them (Fig 3). Other signs observed during examination included 3(10.7%) granny sandy patch (Fig 4) and **4** (14.3%) rubbery papules (Fig 4).

### Prevalence of reported female genital-related symptoms

The most frequent self-reported FGS symptom among study participants was pelvic pain (84.4%) followed by irregular menses (62.5%) and the least reported symptoms were genital bleeding (11.8%) and postcoital bleeding (15.6%) (Table 2).

**Table 2:** FGS reported symptoms among study participants.

FGS symptoms	Outcome	Proportion (%)	95% Confidence interval
**Genital bleeding**	Yes	41 (11.8%)	0.0861–0.1569
	No	306 (88.2%)	
**Post coital bleeding**	Yes	54 (15.6%)	0.1191–0.1981
	No	293 (84.4%)	
**Pelvic pain**	Yes	293 (84.4%)	0.8019–0.8809
	No	54(15.6%)	
**Genital ulceration**	Yes	174 (50.1%)	0.4476–0.5553
	No	173 (49.9%)	
**Genital discharge**	Yes	179 (51.9%)	0.4647–0.5727
	No	166 (48.1%)	
**Irregular menses**	Yes	215 (62.5%)	0.5715–0.6763
	No	129 (37.5%)	

### Risk factors and symptoms associated with female genital schistosomiasis

Table 3 below summarizes the results of the logistic regression model aimed at identifying symptoms and risk factors for FGS using the symptoms and risk factors checklist. After controlling for other factors, participants who reported fetching water in contaminated freshwater sources (aOR:21.8, 95%CI;2.8–171.2, *P*-value = 0.003), reported pelvic pain (aOR:5.3, 95%CI; 1.1–25.916, *P*-value = 0.04) and reported urinary symptoms (aOR:12.19, 95%CI; 1.5–96.3, *P*-value = 0.018 remained independently associated with FGS (Table 3).

**Table 3:** Risk factors and symptoms of FGS among women in Maswa district, Tanzania.

Characteristics	Adjusted OR	*95% CI	∞P-value
**Age**			
Others	1		
25–34	1.777	0.712–4.437	0.2
**Village**			
Isanga	1		
Jija	0.8929	0.061–13.059	0.934
Malampaka	0.4221	0.067–2.661	0.359
**Contact water bodies**			
No	1		
Yes	0.9472	0.102–8.803	0.962
**Swamp farming**			
No	1		
Yes	1.7584	0.266–1.630	0.558
**Fetching water**			
No	1		
Yes	21.78	2.771–171.21	0.003
**Distance from water**			
<10Km	1		
10 – 15Km	2.98	0.2144–38.41	0.426
≥ 15Km	0.199	0.246–1.606	0.130
**Pelvic pain**			
No	1		
Yes	5.296	1.082–25.916	0.04
**Genital ulceration**			
No	1		
Yes	4.216	0.337–52.785	0.265
**Genital discharge**			
No	1		
Yes	2.890	0.510–16.37	0.231
**Irregular menses**			
No	1		
Yes	1.429	0.3069–6.656	0.649
**^Urinary symptoms**			
No	1		
Yes	12.19	1.544–96.303	0.018

^Urinary symptoms -blood in urine, pains during urination, and urine leakage, P-value-Likelihood Ratio Test, * CI- Confidence Interval, °- OR-Odds Ratio, ∞P value- variables adjusted for each other and age, ^1^- fisher’s exact test.

### Sensitivity and specificity of symptoms and risk factors in diagnosing female genital schistosomiasis using digital colposcope as standard diagnostic test

Using the digital colposcope as a standard/reference diagnostic test, the overall sensitivity, specificity, the positive and negative predictive value of symptom and risk factor checklist tool in diagnosing FGS were 85.7% (95%CI; 80.6%- 90.9%), 8.7% (95%CI; 4.6%-12.9%), 15% (95%CI; 9.7%-20.3%) and 76.5% (95%CI; 70.2%-82.7%) among participants who underwent gynecological examination (Table 4). This analysis included only 177 women who underwent colposcopic examination

**Table 4:** Sensitivity and Specificity of symptom and risk factor checklist tool for identifying women suspected to have FGS lesions in Maswa district using colposcope as a reference test.

**Diagnostic test**	**Colposcope results**			
	**Score points**	**Negative**	**Positive**	**Total**
	< 7	13 (76.47%)	4 (23.53%)	17 (100%)
	≥ 7	136 (85%)	24 (15%)	160 (100%)
		149 (84.18%)	28 (15.82%)	177 (100%)
	**Sensitivity**	**Specificity**	**PPV**	**NPV**
Symptom and risk factor checklist	85.71% (95%CI:80.56–90.87)	8.72% (95%CI:4.57–12.88)	15.00% (95%CI:9.74–20.26)	76.47(95%CI:70.22–82.72)

Abbreviations: PPV-Positive Predictive Value, NPV-Negative Predictive Value, CI-Confidence Interval

## Discussion

To our knowledge, this is the first study to assess the sensitivity and specificity of symptoms and risk factors diagnostic tools in Tanzania. There is a lack of gold standard diagnosis for FGS screening and diagnosis. This study has shown that women who scored ≥7 points from the checklist demonstrated high sensitivity but low specificity using a digital colposcope as a reference diagnostic test among 177 women who underwent colposcope and questionnaire. This study also confirms the high prevalence of FGS based on the risk factors and symptoms checklist for participants with score points ≥7 and on digital colposcope in rural Tanzania. UpToDate there is no gold standard for screening and diagnosing FGS. Female genital schistosomiasis diagnosis includes urine microscopy, gynecological examination by speculum/colposcope, a biopsy of genital tissue, and molecular diagnosis of genital samples collected at home or clinic [7,11–13], all of which have challenges. Developing a simple diagnostic algorithm [16] with relevant questions to be used by healthcare workers could help in the identification of at-risk adolescent girls and women, who could then be referred to higher-level facilities for further assessment and diagnosis.

Our study also found out that women who were positive for urogenital schistosomiasis were negative for FGS using the digital colposcope. These results were similar to studies conducted in Zimbabwe [4,5] which report the prevalence of visual FGS using a colposcope for women with no *S*.*haematobium* infection. Other studies have also shown that FGS can occur in the absence of egg excretion in urine [26]. This variation in association in this study population poses the need for an FGS diagnostic tool.

### Risk factors associated with FGS based on the risk and symptoms questionnaires

Importantly, we showed evidence of an association between fetching water, urinary symptoms, pelvic pain, and FGS, after adjusting for confounders. Should this be observed, the symptoms and risk factors that were associated with FGS in our study were also observed in a study conducted in Ghana [27]. This is also concurrent with reports from previous studies showing the association of FGS with sexual reproductive symptoms [3,28]. However, these data should be interpreted carefully as data on fetching water would be more reliable by collecting through observation but most studies have reported on self-reported data [29–31]. Urinary symptoms and pelvic pain were self-reported making results prone to recall bias. There is evidence from previous studies that suggests early treatment of urogenital schistosomiasis could control the FGS morbidity [32]. The WHO recommends annual preventive chemotherapy with praziquantel for people living in schistosomiasis endemic areas with a prevalence of 10% or higher but women out of school environment are not included in mass preventive chemotherapy in these areas [33].

### Prevalence of FGS symptoms

Across our study population, the prevalence of FGS from the checklist and visual colposcope were higher (87% and 15.8%). More than ninety percent of women in our study reported non-use of praziquantel drug and more than half of women reported contact with contaminated water bodies. The most frequent self-reported FGS symptom among study participants was pelvic pain (84.44%) followed by irregular menses (62.50%). Our findings are following community studies conducted in north-western Tanzania and Madagascar which showed that most women reported a high proportion of pelvic pain and irregular menses [9,34,35]. The WHO atlas highlights the importance of considering the diagnosis of FGS for women with urogenital signs and symptoms who contact water bodies in schistosomiasis endemic regions [36]. Women might present with granny sandy patches, homogenous yellow sandy patches, abnormal blood vessels, or rubbery papules. In our study, fourteen women were identified with one or more of these characteristic lesions suggestive of FGS.

The findings of the current study call for preventive chemotherapy in this study population to control FGS among women. Women in these areas are not considered for treatment after leaving primary schools and there is no community mass drug administration in these areas. Therefore, before these women may develop the FGS lesion, they should be part of preventive chemotherapy programs. Early treatment reduces the risk of *S*. *haematobium* eggs lodging in the urogenital system, as it kills the sexually mature Schistosomes and reduces the number of eggs released. Thus, we advocate for women to be included in MDA using praziquantel which in turn will reduce the risk for women to end up with FGS lesions.

In addition, a study done in 2001 showed that colposcopes which are expensive and available in district hospitals may not feasible and available in most rural endemic settings [15]. There is a need to develop alternative cheap diagnostic tools to implement in rural schistosomiasis endemic areas. Recently, field-based molecular testing has been developed and tested in African settings for the diagnosis of FGS [13]. The test gives a promising next stage to be deployed in the African public health system but its cost needs to be evaluated. There is a need to integrate FGS prevention, diagnosis, and management in the national NTD programs since this gynecological disease looks to be prevalent but neglected, underresearched, or underreported in most parts and only a few studies have been conducted in Tanzania [9,26,37]. This calls for the need to integrate FGS screening and diagnosis in the NTD programs.

### Limitations

It should be noted the study was not conducted without limitations. The results may have had more credibility if supporting or complimentary tests were done to enhance the diagnosis of genital schistosomiasis and to rule out other STIs, cervical cancer, or other similar lesions that could mimic FGS. Colposcopy has limited specificity. We ought to have considered the use of molecular methods for FGS which are potentially more specific than colposcopy and allow more comparison of results. Because of resource constraints, no other supporting tests, for example, Schistosoma DNA analysis have been done to support the FGS findings. We also relied on single urine to estimate the prevalence of urinary schistosomiasis. If we had analyzed urine samples on 3 consecutive days, the prevalence of these infections would have been even higher than those that we observed. Nonetheless, the study was conducted in October and November which is low transmission for *S*. *haematobium* infection [38]. Nonetheless, this study had the following strengths and led to conclusions and recommendations below.

### Strengths

Some of the strengths of this study were that confounding was taking account during analysis and study design when we collected for variables (demographic characteristics, risk factors, and symptoms of FGS) and we also had a more than 85% response rate from the study population. This study had good internal validity and with strong evidence of association between symptoms, and risk factors of FGS and FGS using the checklist for diagnosis of FGS. This might be generalizable/show external validity to other sub-Saharan settings among women with FGS-suggestive lesions. However, these results should be interpreted with caution as these results could have resulted due to chance.

### Conclusion and recommendation

This study provides data on the prevalence of FGS, and the sensitivity of FGS diagnostic tools in rural endemic settings. The sensitivity of the symptoms and risk factor checklist for diagnosis of FGS was high but achieved low specificity for women who scored ≥7 using colposcope as a reference diagnostic test. Further studies with large sample size are needed to validate the current tools in areas with different transmission intensity of S. haematobium infection. We recommend mass drug administration to women of reproductive age, and the support and management of FGS patients in the broader health system to be strengthened.

## Supporting information

S1 Information Risk factor and symptom checklist for diagnosis of FGS. (DOCX)
