# Supplementary material for: High sensitivity but low specificity of the risk factors and symptoms questionnaire in diagnosing female genital schistosomiasis among sexually active women with genital lesions in selected villages of Maswa District, North-Western Tanzania
Source: PLoS Negl Trop Dis. 2024 Aug 12;18(8):e0012336. doi: 10.1371/journal.pntd.0012336 (PMC11373800; doi:10.1371/journal.pntd.0012336)
Supplement: S1 Information — (DOCX) [file pntd.0012336.s001.docx]

**FGS SYMPTOMS AND RISK FACTOR CHECKLIST**

This tool should be used by the healthcare worker during consultation of the woman / girl with symptoms of FGS. From the patient complaints, the healthcare worker should tick or circle the point(s) corresponding to the age group, risk factor and symptom of FGS as shown on the table below. The total points should be summed at the bottom of the table.

Table showing grading of FGS risk factors and symptoms.

|  | RISK FACTOR/SYMPTOM | CLASSIFICATION | POINTS | COMMENTS |
| --- | --- | --- | --- | --- |
| 1 | Exposure activities to fresh water | Swimming | 5 |  |
|  |  | Playing | 4 |  |
|  |  | Washing | 4 |  |
|  |  | Bathing | 4 |  |
|  |  | Fishing | 3 |  |
|  |  | Swamp farming | 5 |  |
|  |  | Fetching water | 3 |  |
| 2 | Distance to natural water source(s) e.g. lake, river, stream, creek | <10Km | 5 |  |
|  |  | 10-15Km | 3 |  |
|  |  | ≥15Km | 1 |  |
| 3 | Other sources of water e.g. well, borehole | Yes | 2 |  |
|  |  | No | 0 |  |
| 4 | Genital bleeding | Yes | 2 |  |
|  |  | No | 0 |  |
| 5 | Post coital bleeding | Yes | 2 |  |
|  |  | No | 0 |  |
| 6 | Pelvic pain | Yes | 2 |  |
|  |  | No | 0 |  |
| 7 | Genital ulceration | Yes | 2 |  |
|  |  | No | 0 |  |
| 8 | Genital discharge | Yes | 2 |  |
|  |  | No | 0 |  |
| 9 | Irregular menses | Yes | 2 |  |
|  |  | No | 0 |  |
| 10 | Urinary symptoms  -Blood in urine  - Pains during urination  - Urine leaking | Yes | 2 |  |
|  |  | No | 0 |  |
|  | Patient score points |  |  |  |

**DIAGNOSTIC INTERPRETATION**: The WHO recommends the diagnosis of FGS be considered in all women and girls who present with urogenital symptoms and history of recent contact with fresh water in endemic countries. In this scoring, FGS should be considered in any women who presents with any 1 symptom and any 1 risk factor. Therefore, FGS is:

• Likely if ≥7 points: Send for speculum exam and treatment with Praziquantel

• Very unlikely if <7 points: Investigate for other disease
